# Ebola Virus Disease: international perspective on enhanced health surveillance, disposition of the dead, and their effect on isolation and quarantine practices

**DOI:** 10.1186/s40696-016-0023-6

**Published:** 2016-08-31

**Authors:** Preeti Emrick, Christine Gentry, Lauren Morowit

**Affiliations:** University of Maryland Center for Health and Homeland Security (CHHS), 500 West Baltimore Street, Baltimore, MD 21201 USA

**Keywords:** Ebola, Quarantine, Isolation, Death, Health surveillance, United States, Canada, Australia, Africa

## Abstract

Despite the comparatively few cases of Ebola Virus Disease (EVD) that arose outside of Sierra Leone, Guinea, and Liberia in 2014, public health response partners around the world developed a patchwork of plans and policies to monitor thousands of people exposed to EVD, quarantine suspected cases, isolate confirmed cases, and close borders to prevent further spread of the disease. Deeply affected countries such as Sierra Leone, Guinea, and Liberia, as well as less affected countries such as the United States, Canada, and Australia developed special guidance regarding isolation and quarantine measures for EVD. The massive and well-publicized EVD response highlighted international challenges of public health laws and policies, many of which remain largely unchanged since their implementation. This article examines public health measures, including health surveillance and decedent disposition, and their effects on isolation and quarantine practices in six countries (Sierra Leone, Guinea, Liberia, United States, Canada, and Australia) in context of the 2014–2015 EVD response, and makes recommendations.

## Background

Isolation and quarantine are measures used in public health response in order to control the spread of communicable and infectious diseases. Isolation is the act of separating sick people with a contagious disease from people who are not sick. Quarantine is the separating and restricting of the movement of people who were exposed to a contagious disease in case they become sick [1]. These measures played a prominent role in the EVD response in 2014, and many other factors, such as health surveillance and the disposition of the dead, along played a role in the way isolation and quarantine measures were developed and enforced. This article aims to discuss these policies and interactions during the EVD response.

There are many law articles and studies analyzing the policies and response of the international and domestic community during the EVD response. Each country also had existing laws and policies regarding isolation and quarantine that have a long history behind them. The article takes these sources into account as well as real time accounts and documentaries regarding the EVD crisis.

## Introduction

The 2014–2015 West Africa Ebola Virus Disease (EVD) outbreak is the largest in history. As of June 10, 2016, the World Health Organization (WHO) reported a total of 28,616 cases (suspected, probable, and confirmed) and 11,310 deaths, most of which emerged in Sierra Leone, Guinea, and Liberia [2] (collective population of approximately 290 million people). Nigeria and Mali each reported small numbers of cases, and single cases occurred in Senegal, Spain, Italy, and the United Kingdom. Additionally, the United States reported eight imported cases, including two deaths, and two locally acquired cases in healthcare workers [3].

Despite the comparatively few cases that arose outside of Sierra Leone, Guinea, and Liberia, public health response partners around the world developed a patchwork of plans and policies to monitor thousands of people exposed to EVD, quarantine suspected cases, and isolate confirmed cases to prevent further spread of the disease. WHO developed strategies and policies to combat the spread of EVD that rejected blanket travel bans and emphasized education, but ultimately WHO lacks enforcement authority [4, 5] to ensure the uniform implementation of its recommendations or a seamless international response. The varied, massive, and well-publicized response highlighted challenges internationally in public health laws and policies, many of which remain largely unchanged since their implementation. This article examines public health measures, including health surveillance and decedent disposition, and their effects on isolation and quarantine practices in six countries (Sierra Leone, Guinea, Liberia, United States, Canada, and Australia) in context of the 2014–2015 EVD response, and makes recommendations.

## Review

### Survey of international EVD response

Guinea, Liberia, and Sierra Leone:

For me, the worst is quarantine: it means prison. Can you imagine? There is no war but men with guns and uniforms stand outside the homes of your friends. One day, there were soldiers outside my own house.

Bintu Sannoh, a Sierra Leonean on forced quarantines [6]

The constitutions of Guinea, Sierra Leone, and Liberia allow for a wide range of emergency response measures to protect the public’s health during emergencies [7]. Though all three constitutions grant certain public rights, such as freedoms of assembly and association, only Guinea’s constitution explicitly preserves those rights during declared public emergencies [8]. During the EVD outbreak, these countries’ emergency declarations revised their legal landscapes to permit a broader scope of public health and enforcement measures, particularly in three areas: enhanced health surveillance, disposition of the dead, and isolation and quarantine practices.

#### Enhanced health surveillance

The EVD outbreak remained rampant in West Africa for 6–8 months before actual surveillance was launched in order to monitor the spread of the disease [9]. While internationally most countries carried out some level of EVD screening and monitoring, Guinea, Sierra Leone, and Liberia eventually implemented mandatory health checkpoints and house-to-house searches to conduct contact investigations, and developed and enforced strict penalties, including jail time, for those violating public health orders [7].

The implementation of these health surveillance efforts, particularly resource heavy functions such as contact investigations, suffered due to inadequate investigation teams, health service availability, and sharply rising death tolls. The lack of established surveillance, early warning systems, and initial misdiagnosis of EVD cases contributed to the scope of the outbreak.

#### Disposition of the dead

All three countries also required specific methods of death reporting and disposition. Liberia required cremation of EVD victims, while Guinea and Sierra Leone mandated all deaths be reported and restricted transportation of decedents. These public health orders were enforced with fines, quarantines, and even jail time. In Guinea, for example, six people were prosecuted for violating the country’s emergency declaration by transporting a decedent EVD victim in a taxicab [10].

#### Isolation and quarantine practices

In addition to enhanced surveillance, Guinea, Sierra Leone, and Liberia instituted a range of isolation and quarantine practices, though many were implemented months after the EVD outbreak began. Policy in Liberia and Sierra Leone was to quarantine households with an exposed, confirmed, probable, or suspected case for up to 21 days even without displaying symptoms [11]. Two negative lab tests from the original suspected case were required to clear quarantine [8]. Quarantine and isolation measures were strictly enforced by military and law enforcement.

All three countries’ emergency declarations required closures of borders and certain public spaces, such as schools and markets [7]. Liberia and Sierra Leone banned mass gatherings and closed government offices. In response to high prevalence in certain neighborhoods, all three countries mass quarantined portions of their population based on geographic location rather than exposure or symptomology. Sierra Leone instituted a 3 days lockdown in September 2014 during which all residents—regardless of exposure—were required to remain in their residences [7]. Guinea isolated the population in areas with more than a 70 percent infection rate using police and military assets, while Liberia quarantined West Point, one of the country’s poorest and most densely populated neighborhoods [7].

USA:

They would see pictures of West Africans, be they in Liberia, Sierra Leone or Guinea – lying on the street, bodies there not getting picked up – and they said, ‘Oh my God, is this what’s going to happen in the U.S.?’

Dr. Anthony Fauci, Director of the National Institute of Allergy and Infectious Disease [12]

Much like the constitutions of Guinea, Sierra Leone, and Liberia, the authority of federal and state governments in the United States derives from the United States Constitution. While both federal and state governments have isolation and quarantine powers, state governments are the main authority for implementing public health measures to protect the health, safety, and welfare of persons within their borders [5]. Under this system of federalism, state public health measures may clash with federal guidelines and policies.

#### Enhanced health surveillance

Surveillance measures focused on travelers, including many returning health care workers, from West Africa. According to the 2015 Federal Emergency Management agency (FEMA) National Preparedness Report, U.S. Customs and Border Protection (CBP) screened 6846 total passengers arriving from affected countries [13]. The Centers for Disease Control and Prevention (CDC) issued guidance regarding the recommended health surveillance measures based on four categories of risk—high risk, some risk, low (but not zero) risk, and no identifiable risk [14]. These categories determined the type and level of health monitoring and movement restrictions state and local health departments should implement during the 21 day incubation period of the virus [14]. Most health care workers returning from West Africa were considered to have some risk, which required direct active monitoring, including daily monitoring of symptoms and assessment of any potential travel, as well as a potential restriction of movement [14, 15].

#### Disposition of the dead

Due to the high risk of transmission involved in postmortem care settings, the CDC outlined protocols for handling EVD-related deaths in the United States. This guidance directs trained personnel not to do the following when disposing of a body infected with EVD: clean or wash, embalm, remove any inserted medical equipment from the body, or perform an autopsy [16]. The first EVD-related death in the United States required that the decedent’s body remain unwashed, wrapped in a plastic shroud, and then placed into a zippered leak-proof bag. Ultimately, the transportation of the body was coordinated by the CDC and local transportation authorities to a mortuary for cremation [17].

#### Isolation and quarantine practices

The health surveillance measures put in place by most state and local public health entities required some measure of quarantine, and confirmed cases of EVD were isolated [18]. While the CDC guidance recommended against forced or mass isolation and quarantine orders to avoid violating civil liberties, under the current framework of public health laws, states were free to follow the CDC guidance or implement more stringent policies in place [19–21]. New York and New Jersey (and many others [22]), for example, enacted far stricter public health measures than those recommended by the CDC, requiring that all those returning from West Africa with any level of EVD exposure be placed in a mandatory quarantine, regardless of symptoms or the lack thereof [23, 24]. The case of Kaci Hickox, a healthcare worker who volunteered in West Africa, illustrated the civil liberties issues that may arise with forced public health orders. Eventually, the state court in Hickox’s home state of Maine ruled against forced quarantine because the restriction of her freedom of movement was not warranted in accordance with CDC guidance regarding disease transmission prevention.

Australia and Canada:

The spirit of IHR is that the measures need to be commensurate and there shouldn’t be any restrictions in international travel if not recommended by an emergency committee.

Dr. Isabelle Nuttall, Head of the WHO’s Global Capacities Alert and Response department, on blanket travels bans enacted [25]

Australia and Canada have similar medical treatment and infrastructure as the United States; nonetheless, the same fears about EVD occupied both countries and informed public health policies during the height of the EVD outbreak. While the United States had confirmed EVD cases, Canada and Australia had none [26, 27].

#### Enhanced health surveillance

Under the Public Health Agency, Canada issued guidelines regarding the monitoring and movement of people travelling from West Africa. These guidelines included two main categories—travelers without symptoms and travelers with symptoms [28]. Travelers without symptoms were grouped into high risk and low risk groups, depending on whether there had been direct contact with EVD patients and the amount of personal protective gear worn, and were advised to self-monitor and report any planned travel if low risk, or be monitored for symptoms and self-isolate if high risk [6]. Humanitarian workers were placed in their own category similar to the low risk, with the caveat that self-isolation would be required if, for example, there was a known breach in their personal protective equipment [28]. Public health officials and aid groups who were fearful of a stricter policy welcomed this federal policy as it provided flexibility [29], and provinces like Ontario followed the federal guidelines [30].

#### Isolation and quarantine practices

Australia and Canada’s isolation and quarantine measures focused on entry into the country. These flexible and reasonable guidelines for humanitarian workers stood in contrast with Canada’s actions concerning border control. In contrast to WHO guidelines, Canada stopped processing new and pending visa applications from Sierra Leone, Guinea, and Liberia, and applications of those who were in the above countries 3 months prior to the application being received [31–33]. These measures effectively closed the Canadian border and were arguably unnecessary as the public health risk to Canada was very low [34].

Australia was the first developed country to close its borders in response to EVD [35]. Under section 51(ix) of the Australian Constitution, the Commonwealth has the power over the states regarding quarantine. The Biosecurity Bill 2014 was introduced during the EVD outbreak in West Africa, and it aimed to prevent the spread of diseases such as EVD. Furthermore, the Biosecurity Bill grants a health department official the authority to force anyone with signs or symptoms of a listed disease to practice voluntary isolation or face arrest [36]. On October 28, 2014, Australia suspended visa assessments for applications from citizens from Sierra Leone, Liberia, and Guinea, cancelling non-permanent or temporary visas [37, 38].

In addition, Australia suspended its humanitarian program and stopped accepting West African refugees [38, 39]. Those with permanent visas who had not yet come to the country were asked to submit to a mandatory 21 day quarantine period once they arrived, regardless of their exposure history [38, 40]. Australia refused to send health workers to support the EVD response in Africa, citing the long distance and travel between the affected areas and Australia would make it very difficult for the evacuation of such workers if they became infected with EVD [41, 42].

### Survey comparison

**Table:** 

	Guinea	Liberia	Sierra Leone	United States	Canada	Australia
EVD cases confirmed/present	Yes	Yes	Yes	Yes	No	No
Surveillance methods	Yes	Yes	Yes	Yes	Yes	
Border control measures	Yes	Yes	Yes	No	Yes	Yes
Isolation and quarantine measures	Yes	Yes	Yes	Yes	Yes	Yes
Disposition of dead measures	Yes	Yes	Yes	Yes	N/A	N/A
Military enforcement	Yes	Yes	Yes	No	No	No

## Conclusion and recommendations

Western countries are creating mass panic which is unhelpful in containing a contagious disease like Ebola.

Ofwono Opondo, Ugandan government spokesman, in response to Australia’s visa suspension policy [35]

During the EVD crisis, governments implemented public health laws with mixed results. The delayed implementation of comprehensive, EVD-specific public health measures in West Africa required that the measures themselves be implemented on a larger, more extreme scale. Just as EVD causes long-term health effects in survivors, strict public health orders, such as mandatory isolation and quarantine, business and school closures, and travel bans, have immediate and lasting consequences on the affected individuals and communities [43]. For instance, the use of mass quarantine immediately restricted people’s rights to liberty and freedom and created large-scale food and shelter scarcity and civil unrest. School attendance remains very low [43]. Health service delivery has seen a 23 % decrease, and other essential services like water and sanitation experience continued disruption [14]. Additionally, EVD survivors and their families face discrimination in their communities; some survivors report having to move [28]. While the effects would vary from jurisdiction to jurisdiction, possible long-term ramifications of similar public health measures must be considered when developing response policies and procedures.

In the United States, the well-established stories of the ten confirmed EVD cases as well as Kaci Hickox demonstrate that while public health laws and policies are in place, influences such as political realities, fear, and unclear jurisdictional delineation can create uneven and haphazard public health protection. Similar to the United States’ divergence from WHO guidelines, Australia and Canada implemented policy seemingly based on public reaction and fear. All three countries implemented response measures similar in many ways to the measures enacted in the worst affected countries, despite the lower incidence and prevalence of EVD.

While the principles of sovereignty, and in the United States, the police power, certainly grant jurisdictions the authority to implement a wide range of measures to protect public health and prevent transmission of diseases, including mandatory isolation and quarantine orders, blanket travel bans, and other restrictions, these powers should be implemented based on the best knowledge and practices of medical science, not public panic. The key to addressing a global public health crisis like the EVD outbreak is adopting a uniform, evidence-based approach and ultimately controlling the crisis at its source. Since laws regulating public health emergencies and orders are not frequently activated except in large or well-publicized incidents, and often have not been updated to reflect evolving best practices and developments in technology, reexamination of these laws and regulations is critical to avoid violations of civil liberties and long-term ramifications, as well as the undermining of ongoing public health and humanitarian operations.

A few recommendations for future public health emergencies include:

1. Governments should employ the least restrictive means necessary—on the basis of the best available scientific evidence—in implementing isolation or quarantine measures
2. There should be increased transparency and the promotion of communication between centralized agencies/organizations and localities in order to better streamline policies and public health surveillance.
3. All governments after a public health emergency and before the next emergency should make a better determination of the national hospital capacity to handle infectious disease patients and try to address the gaps found.
4. Governments should review their laws and authorities for quarantine and isolation and make any necessary changes to strengthen just enforcement.

## Abbreviations

CBP: U.S. Customs and Border Protection
CDC: Centers for Disease Control and Prevention
CHHS: University of Maryland Center for Health and Homeland Security
EVD: Ebola Virus Disease
FEMA: Federal Emergency Management Agency
WHO: World Health Organization
